# Treatment of patellofemoral osteoarthritis with nasal chondrocyte-based engineered cartilage implantation in a randomised, controlled, multicentre phase II clinical trial: protocol for a randomised controlled trial

**DOI:** 10.1136/bmjopen-2025-106140

**Published:** 2025-08-21

**Authors:** Sarina Seitz, Gyözö Lehoczky, Anke Wixmerten, Corina Schuster-Amft, Sylvie Miot, Kristin Shrestha, Sabine Schaedelin, Ivan Martin, Marcus Mumme

**Affiliations:** 1 Associate of the Faculty of Medicine, University of Basel, Basel, Switzerland; 2 Research Department, Reha Rheinfelden, Rheinfelden, Switzerland; 3 Department of Biomedicine, University of Basel, University Hospital Basel, Basel, Switzerland; 4 Sports Orthopaedics, University Children’s Hospital Basel (UKBB), Basel, Switzerland; 5 School of Engineering and Computer Science, Bern University of Applied Sciences, Biel, Switzerland; 6 Department of Sport, Exercise and Health, University of Basel, Basel, Switzerland; 7 Department of Clinical Research, University Hospital Basel, Basel, Switzerland; 8 Orthopaedic Surgery and Traumatology, University Hospital Basel, Basel, Switzerland; 9 Sportclinic Zürich, Klinik Hirslanden, Zurich, Switzerland

**Keywords:** Knee, SURGERY, Adult orthopaedics

## Abstract

**ABSTRACT:**

**Introduction:**

Knee osteoarthritis often starts in the patellofemoral compartment of the knee and is diagnosed in about 39% of people with knee pain aged above 30 years. Patellofemoral osteoarthritis plays a crucial role in the reduction of quality of life and in the rise of healthcare costs. There is still no consensus for treatment recommendation for isolated patella-femoral osteoarthritis in clinical guidelines. Current therapeutic approaches are limited to pain management, alleviation of symptoms or total knee replacement. Nasal chondrocyte tissue-engineered cartilage (N-TEC) has already been successfully introduced in clinical studies phase I and II for the treatment of focal cartilage lesions and in pilot studies in osteoarthritis patients.

**Methods and analysis:**

A randomised controlled trial involving 75 patients with patellofemoral osteoarthritis from nine different clinical centres in Switzerland, Germany and Croatia is being conducted to evaluate the effectiveness of N-TEC implantation compared with standard treatment with platelet-rich plasma (PRP). In the intervention group, an autologous nasal cartilage cell-derived graft is implanted into the cartilage defects of the patella and/or trochlea during an open surgical procedure. The control group receives three PRP injections at weekly intervals. The primary outcome is the mean Knee Injury and Osteoarthritis Outcome Score Pain Change from baseline to 24 months between groups. Secondary outcomes, including patients’ self-assessed questionnaires, X-ray and MRI scans, physiotherapeutic assessments and safety, will be assessed and compared between the intervention and control group. In addition, the study is complemented with a health-economic evaluation to establish the intervention’s value for money and impact on productivity in working-age individuals. The planned duration of the study is 4 years including baseline and follow-up measurements at 6, 12 and 24 months.

**Ethics and dissemination:**

All centres involved in the implementation of the intervention have obtained approval from their respective competent ethics committees. This includes approval from the following ethics committees: Ethics Committees of North-Western and Central Switzerland (EKNZ): 2024–00075 (associated ethical committees: Cantonal Ethics Committee Bern, Cantonal Research Ethics Commission Geneva (CCER), Cantonal Ethics Committee Ticino, Cantonal Ethics Committee Zurich). The EKNZ covers several cantons in Switzerland, including Basel. The site in Lugano falls under the Cantonal Ethics Committee Ticino. Ethics Germany according to CTIS: 2023-508640-21-00 (Medicinal Ethical Commission of the Julius-Maximilians-University Wuerzburg, Ethical Commission of the Albert-Ludwigs-University Freiburg) and Central Ethical Committee Croatia, Republic of Croatia Ministry of Health: 2023-508640-21-00. The Swissmedic reference number is 701788.

Prior to participation, all participants must have signed informed consent. Study information will be disseminated via hospital websites, newsletters and an open-access publication of the protocol. Results will be published in peer-reviewed journals, presented at national and international conferences and shared with the public.

**Trial registration number:**

ClinicalTrials.gov Registration No.: NCT06163573; Registration number CTIS: 2023-508640-21-00.

**STRENGTHS AND LIMITATIONS OF THIS STUDY**

- Novel therapeutic approach enabling cartilage transplantation in osteoarthritic joints for the first time in a clinical study.
- Use of tissue-engineered nasal cartilage allowing treatment of challenging defects, including kissing lesions.
- High-level evidence design with the potential to extend treatment recommendations for isolated patellofemoral osteoarthritis.
- Inability to blind patient groups due to non-surgical comparator, increasing risk of performance and detection bias.
- Potential patient perception of greater benefit from surgical intervention, introducing bias in self-reported outcomes.

## Introduction

### Background and rationale {6a}

 Approximately 39% of patients with knee pain are diagnosed with some form of patellofemoral osteoarthritis (PFOA), and around one-fifth of these individuals present with isolated PFOA.^1 2^ Recent studies suggest that knee osteoarthritis (OA) is more likely to initially affect the patellofemoral joint, subsequently progressing to combined OA in individuals displaying early symptoms of knee OA.^3^ Specifically, the study demonstrated that approximately 50% of participants with isolated PFOA developed combined OA within 2 years, and two-thirds progressed to combined OA within 5 years.^3^ These findings emphasise the significance of isolated PFOA in the natural history of knee OA and highlight the need for regenerative treatment strategies to alleviate pain, improve symptoms and potentially delay disease progression to combined (tri-compartmental) knee OA.

Current treatment options for isolated PFOA encompass various approaches aimed at symptom management (eg, physiotherapy, bracing, intra-articular hyaluronic acid (HA) injections, arthroscopy), restoration of biomechanics (eg, patellar remodelling, lateral release and tibial tuberosity osteotomy) and, ultimately, prosthetic implantation (eg, patellofemoral or total joint replacement).^2^ However, due to a lack of high-quality studies, none of these treatments is considered a gold standard for isolated PFOA under evidence-based criteria, and no disease-modifying therapies are currently available.^2^ In the early stages of OA, non-pharmacological interventions are typically employed to focus on pain relief and improved knee function.^4^ Pharmacological treatments (anti-inflammatory drugs) may be used concurrently and are employed in later stages to manage pain,^4^ though these drugs are associated with known side effects (eg, drug toxicity), and their long-term efficacy remains variable or undetermined.^5^ Intra-articular treatments have been found to alleviate symptoms, but their long-term efficacy is still under scrutiny,^4^ with the effects of viscosupplementation typically lasting between 6 and 12 months.^6 7^ When other treatments have proven ineffective, surgical joint replacement (total knee arthroplasty or patellofemoral arthroplasty) may be considered. Despite its apparent effectiveness, no randomised controlled trials (RCTs) have been conducted to validate these procedures.^2^ Knee replacement represents a significant intervention with irreversible consequences due to the partial removal of knee structures, and it is not a permanent solution. Moreover, 15%–20% of patients are dissatisfied with the outcome following knee replacement, and particularly in younger patients (under 60 years of age), the risk of revision surgery, which is associated with reduced efficacy, is increased by 20% (women) to 35% (men), with half of all revisions occurring within the first 5-years post-implantation.^8^

Crucially, none of the conventional approaches address tissue regeneration, whereas a regenerative treatment option would be vital to prevent or at least delay the need for artificial joint replacement. Several molecules are currently under clinical investigation for their disease-modifying effects in OA, with the aim of not only slowing degradation but also promoting the regeneration of articular cartilage.^9^,^16^ Most of these molecules are administered via intra-articular injections. While some of those do have a positive effect on the cartilage, they do not improve symptoms (Sprifermin13^9^) or they can build up cartilage for femoral defects, but not for patella defects (LNA04315^15^). In summary, the expected effects of these drug therapies are moderate symptom relief with little or no superiority to placebo and little cartilage structure preserving or building effects. Because the potential of these drugs lies in the long-term structural maintenance of articular cartilage, such therapy should possibly be started in the early stages of OA. However, it is not possible to claim an actual remission of the disease and repair of extensive cartilage lesions.

Cell-based regenerative therapies are a promising alternative approach, but none of the ones available on the market for focal cartilage defects is applicable for knee OA or kissing lesions in PFOA and most of them are not recommended for the elderly (above 55 years). All available cell-based therapies are based on the use of autologous articular chondrocytes (AC), whose proliferation capacity and postexpansion differentiation potential are known to have a high variability and to decrease with the age of the patient.^17^ The age dependency of AC properties is a critical issue, since OA often affects patients above 55 years. Moreover, it is challenging to harvest ‘healthy’ AC even from the macroscopically unaffected area of a diseased OA knee, considering that the properties of the harvested chondrocytes might be altered in OA joints.^18^ Often neither the cell starting number nor the expansion capacity of AC is sufficient for implantation in large surface areas commonly affected in OA.^18^ Therefore, we propose to use an innovative cell source, nasal chondrocytes (NC), to engineer tissue cartilage graft for the regenerative treatment of PFOA.

### Objectives {7}

#### Overall aim and objective

The study aims to examine whether the implantation of NC tissue-engineered cartilage (N-TEC) will yield superior clinical outcomes compared with the comparator, injection of platelet-rich plasma (PRP). Furthermore, the study should help to verify the regenerative nature and disease-modifying properties of the treatment.

#### Primary objective

This phase II trial primarily aims to determine whether implantation of N-TEC will lead to a superior clinical outcome, as assessed by the change in Knee Injury and Osteoarthritis Outcome Score (KOOS) Pain from baseline to 24 months, as compared with the control group. The comparison between groups will enable to evaluate if the implanted graft demonstrates greater efficacy than the comparator after 24 months.

#### Secondary objectives

Secondary objectives aim (1) to compare the development of the patients’ recovery over time between groups and (2) to determine the integration of the graft, the maturation of the repair tissue and the progression or remission of the disease. In addition, the regenerative potential of the treatment will be assessed by evaluating cartilage regeneration. Treatment failures are also of interest for the evaluation.

#### Safety objectives

A further objective of the study is to examine the safety of implanting N-TEC.

#### Health economic objectives

The intervention strategy induces substantial costs. Given strained healthcare resources, new treatment strategies do not only need to show favourable clinical properties but also a favourable or at least acceptable cost–benefit ratio. The study is, therefore, complemented with a health-economic evaluation (assessment of costs and cost-effectiveness) to establish the intervention’s value-for-money and impact on productivity in working-age individuals.

### Trial design {8}

This study is a randomised, open, controlled, parallel-group, prospective superiority trial (phase II) to evaluate the efficacy of implantation of N-TEC versus injection of PRP for the treatment of PFOA. A total of 75 patients, suffering from symptomatic PFOA (Iwano grade 1–3) will be randomised one to one to either N-TEC or to the active comparator control group (Injection of PRP). The experimental group will receive the implant, while the control group will undergo three intra-articular injections over a 3-week period. Both groups will subsequently participate in an 18-week physiotherapy programme and will be followed up for 2 years.

**Figure 1:**
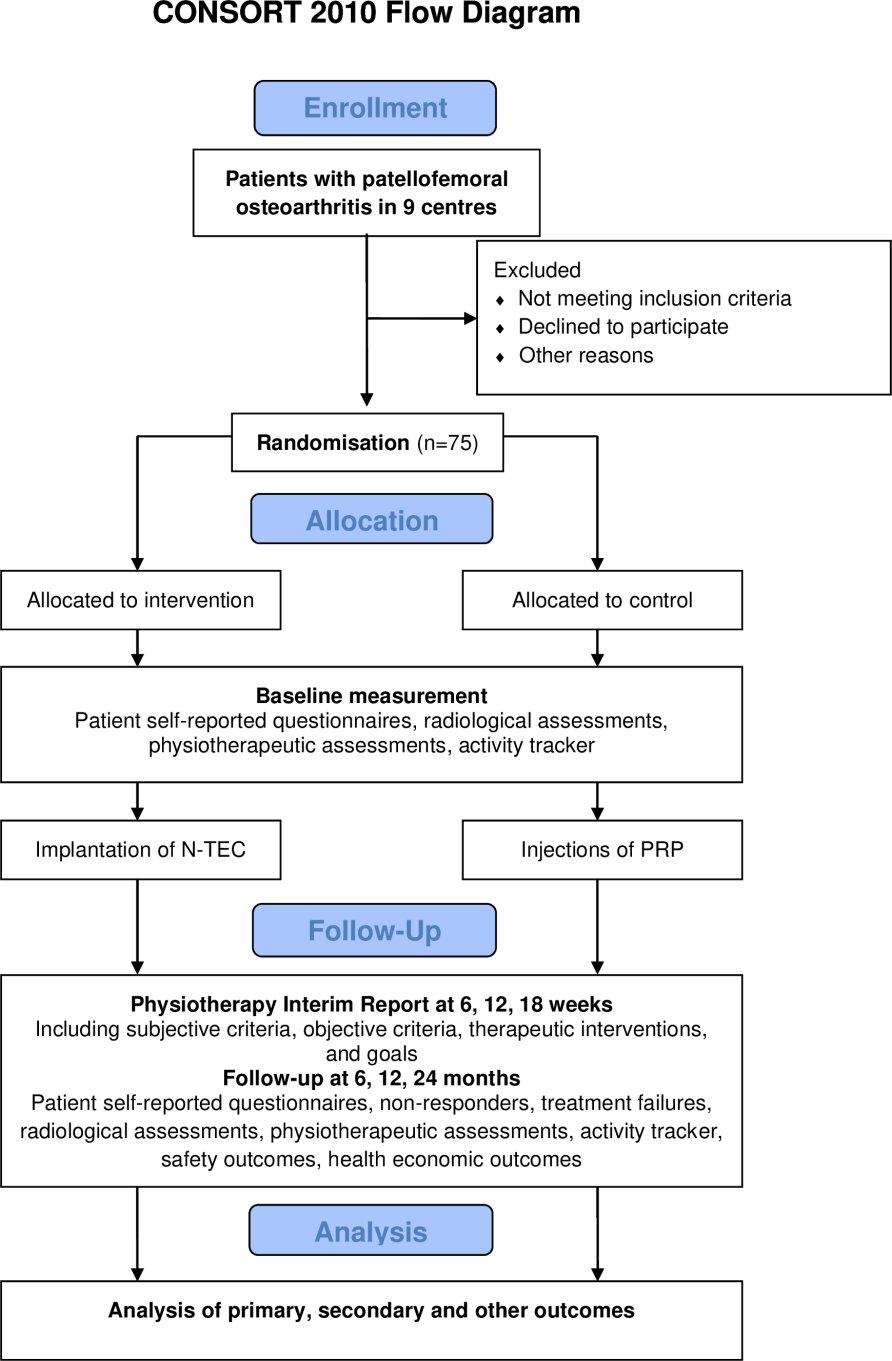
Study design flowchart (adapted from the Consolidated Standards of Reporting Trials (CONSORT) 2010 flow diagram). N, number; N-TEC, nasal chondrocyte tissue-engineered cartilage; PRP, platelet-rich plasma.

The adapted Consolidated Standards of Reporting Trials (CONSORT) 2010 study design flow diagram provides an overview of the study procedures, including key phases such as participant recruitment, treatment and follow-up assessments (figure 1).^19^

## Methods: participants, interventions and outcome

### Study setting {9}

This study is an international multicentre study involving six centres in Switzerland, two centres in Germany and one centre in Croatia. Patients will be enrolled at these nine clinical centres in Basel (CH, two sites), Zürich (CH, one site), Geneva (CH), Lugano (CH), Bern (CH), Freiburg (DE), Wuerzburg (DE) and Zagreb (HR).

### Eligibility criteria {10}

#### Inclusion criteria

- Patient age is ≥18 and ≤65 years at time of screening.
- Symptomatic PFOA grade 1–3 according to the Iwano classification.
- Chondropathy grade 3–4 according to the International Cartilage Repair Society classification of the patella, trochlea femoris or both.
- Baseline score of <60 on the KOOS Pain subscale.
- Free range of motion of the affected knee joint or ≤5° of extension loss and minimum 125° flexion.
- Patient is willing and able to give written informed consent to participate in the study and to comply with all study requirements, including attending all follow-up visits and assessments and to complete postoperative rehabilitation regimen.
- Minimum values for women: haemoglobin 120 g/L, platelets 150 G/L, International Normalized Ratio (INR) <1.3.
- Minimum values for men: haemoglobin 140 g/L, platelets 150 G/L, INR <1.3.
- Non-surgical standard of care options except for PRP have been exhausted.

#### Exclusion criteria

- Patient is the investigator or any subinvestigator, research assistant, pharmacist, study coordinator, other staff or relative thereof directly involved in the conduct of the protocol or in a dependency or employment with the sponsor.
- Patient is unable to understand the patient information.
- Patient is unable to undergo MRI.
- Prior surgical treatment of the target knee within 12 months (note: prior diagnostic arthroscopy with debridement and lavage is acceptable within 12 months).
- Radiologically apparent degenerative joint disease of the tibiofemoral joint as determined by X-ray (Kellgren and Lawrence grade >2) or MRI or pain in the tibiofemoral joint as assessed by clinical examination.
- Patient has excessive varus or valgus deformity (>5°).
- Patient had a patellar dislocation in the afflicted knee in the last 2 years.
- Patient has a symptomatic meniscus lesion (or removal exceeding 1/2), as indicated by clinical examination (joint line tenderness and McMurray test positive) and MRI.
- Patient has a body mass index >35 kg/m^2^.
- Patient has chronic rheumatoid arthritis and/or infectious arthritis.
- Any concomitant painful or disabling disease of the spine, hips or lower limbs that would interfere with evaluation of the afflicted knee.
- Patient has a known immunological suppressive disorder or is taking immunosuppressives.
- Patient had any intra-articular injections into the affected knee within the last 3 months before baseline visit.
- Instability of anterior, posterior collateral ligaments.
- The patient has an HIV/AIDS infection (regulatory requirement).
- The patient has an acute Treponema pallidum (syphilis) infection (regulatory requirement).
- The patient has an active hepatitis B or C infection with verified antigens. Patients with a cured hepatitis B or C infection and/or verified antibodies are not excluded (regulatory requirement).
- Patient is pregnant, breastfeeding or anticipates becoming pregnant within 24 months after surgery.
- Patient is currently participating or has participated in any other clinical study within 3 months prior to the screening visit.
- Patient has known current or recent history of illicit drug or alcohol abuse, or dependence defined as the continued use of alcohol or drugs, despite the development of social, legal or health problems.
- Patient has any other condition, which, in the opinion of the investigator, would make the patient unsuitable for the study.
- Any known allergies, especially for porcine collagen, penicillin or streptomycin (manufacturing).
- Patients at increased anaesthesiological and surgical risk (eg, known or predicted difficult airway, myocardial infarction <60 days prior to surgery).
- Patients with increased bleeding risk (eg, coagulopathies).
- Patients on anticoagulants whose anticoagulant therapy cannot be interrupted as appropriate to the given agent(s) and underlying condition.
- Patients with any active infections.

### Who will take informed consent? {26a}

Before enrolment in the study, the patient will receive information in an oral and written manner by the PI of the respective centre or a trained delegated person and will be given time to discuss the treatment options and study participation with relatives or close persons. Patients will not be coerced into participating and will not suffer any disadvantage in treatment by declining to participate. On signing the informed consent (see online supplemental material ‘PFOA II_patient information and informed consent_V02’), the patients will be investigated, to see if they are eligible for the clinical trial and meet the inclusion and exclusion criteria defined in the study protocol. If the patient complies with the eligibility criteria, they will be assigned to a treatment group, noted in the enrolment log and given a Patient-ID.

### Additional consent provisions for collection and use of participant data and biological specimens {26b}

Data, including genetic information and biological samples obtained in the context of this study, will only be used for future research if the participant provides explicit, written consent. The participant information sheet includes a dedicated section outlining this option, which requires a separate signature. Declining this optional consent will not affect participation in the current study. In such cases, data and samples will be excluded from any future research use.

## Interventions

### Explanation for the choice of comparators {6b}

To demonstrate the clinical efficacy and regenerative nature of the treatment in a high-level evidence clinical study, a control arm needs to be defined. Typically, this is based on standard-of-care, which in the case of OA is not possible, as only treatments for symptoms are available. We objectively assessed different possible comparators for safety and efficacy and discussed them with clinical experts in the field of OA and cartilage repair. Based on the considerations above, we selected autologous leucocyte poor PRP injections as active comparator. Although HA is widely used and recommended for OA treatment, many patients have already tried this treatment. Importantly, recent systematic reviews and meta-analysis comparing PRP to HA injections have been shown to lead to a higher improvement in pain and function in knee OA in the PRP group.^20^ The overall improvement of patients receiving PRP was higher than those of the HA group, 81% versus 38%.^20^ This improvement may be linked to the anti-inflammatory and anabolic effects of PRP, while HA mainly reduces pain by enhancing joint lubrication, which wears off with time.^7 20^ PRP is also reported to delay total knee replacement.^21^ Moreover, data indicate that PRP treatment offers the best benefit/risk balance and has a more favourable safety profile as compared with non-steroidal anti-inflammatory drug also in long-term use and may be associated with a gain in quality-adjusted life years and reduced need for other OA treatments.^6 22 23^ The clear disadvantages are that PRP is a non-surgical comparator, preventing blinding of the study for ethical reasons, and that this treatment has in most studies only been followed up for up to 1 year. To also account for the short-term benefit typical for PRP treatment, follow-ups will be performed also at 6 and 12 months; however, longer follow-up times are very important in the field of OA studies. Based on the previous studies, first effects for the surgical approach are also seen after 12 months. A comparison with prosthetic treatment does not seem feasible as patients with mild PFOA (Iwano grade 1–2) would not qualify for a prosthesis.^24 25^

### Intervention description {11a}

#### Experimental group

The investigational medicinal product (IMP) is N-TEC. The active components are expanded human autologous NC and cartilage matrix proteins produced by the cells. The IMP is considered a combined ATMP as a medical device (Chondro-Gide^®^) is an integral part of the product. N-TEC is generated using autologous NC isolated by enzymatic digestion from a 6 mm diameter biopsy of the nasal septum and expanded for 2 weeks in monolayer. After expansion, cells are seeded on a 40×50 mm collagen membrane (Chondro-Gide^®^) at a cell density of 4.2 million cells/cm^2^ and cultured for two additional weeks to allow for production of extracellular matrix by the cells. There is a total of 4 weeks between the nasal biopsy and the implantation of the cartilage graft in the knee. After manufacturing, the engineered tissue is sent to the respective clinic, cut and shaped according to the defect size by the surgeon and immediately implanted using bioresorbable sutures for fixation until the graft heals in. Additional procedures to treat risk factors of patellofemoral cartilage damage such as treatment of patellar instability with medial patellofemoral ligament procedure or high patella configuration with tibial tuberosity osteotomy are allowed. In case of deep osteochondral lesion, bone grafting is allowed to reconstruct subchondral bone without need for additional preparation. Any unused part of the generated graft will be discarded according to hospital regulations. Since this is a one-time treatment, no dosing schedule or treatment periods apply.

#### Control group

The leucocyte-depleted PRP is prepared and injected once per week during 3 consecutive weeks in the knee using the Arthrex ACP^®^ system. Approximately 15 mL venous blood is taken from the patient and processed according to the instructions of the Arthrex ACP^®^ system, resulting in 5±1 mL of PRP. The PRP is afterwards injected completely into the knee joint under sterile conditions.

For both groups, follow-up visits will be carried out at 6 weeks, 6, 12 and 24 months. Besides physiotherapy planned for 18 weeks, no further treatment is planned after surgery or PRP injection.

### Criteria for discontinuing or modifying allocated interventions {11b}

Patients can be excluded from the study by the respective PIs at any point for the following reasons:

- Medical reasons where a continuation of the trial would jeopardise the health of the patient.
- Withdrawal of informed consent (final medical follow-up mandatory for patient safety).
- Non-compliance with the required procedures as stated in the patient information and informed consent or refusal of the follow-up examinations, which are necessary to assess the safety and efficacy of the treatment.
- Abortion of the clinical study.
- Contamination of the IMP during manufacturing.
- IMP manufacturing not successful.

For the experimental surgical approach, dose modifications are not possible. For the control group, no dose modifications are planned.

### Strategies to improve adherence to interventions {11c}

Since the treatments (implantation of N-TEC, injection of PRP) will be done within hospital settings, there is no risk of non-compliance. All clinical procedures will be standardised through training sessions (both theoretical and practical) and written standard operating procedures. In the event of missed follow-up appointments, surgeons will contact patients to reschedule the visit. Patients will complete questionnaires online up to 2 weeks prior to the visit or directly at the time of follow-up using an electronic case report form (eCRF).

As physiotherapy plays a critical role in clinical outcomes, direct communication from the study physiotherapist via email or telephone with patients as well as therapists is essential to monitor adherence throughout the 24-month study period and ensure compliance. Therapists will be informed of their patients’ study participation via email and provided with written information on standardised therapy recommendations according to group allocation. Additionally, they will receive a brief online training session conducted by the lead physiotherapist. A rehabilitation dossier, including exercise programmes, will be provided to optimise implementation. At 6, 12 and 18 weeks, physiotherapists will submit a brief interim report detailing therapy progress.

### Relevant concomitant care permitted or prohibited during the trial {11d}

Physiotherapy will be given to the patient after implantation to restore mobility and muscle formation as part of the study protocol. Patients need to follow a rehabilitation programme to ensure proper reintegration into daily activities, work and sports and leisure. This process has to be accompanied by physiotherapists. Physiotherapy is considered to have a significant impact on the patients’ recovery process.

The main aims of the programme will focus on pain reduction, muscle strengthening (knee and hip muscles), proprioceptive training/stabilisation, optimising neuromuscular function and patient education. Rehabilitation programmes for both arms will aim at being as similar as feasible, both in regimes applied and in measures for adherence. However, during the initial phase (12 weeks), the therapy will differ as follows:

#### Experimental group (N-TEC)

The rehabilitation programme is designed to protect the implanted graft during the early postoperative phase. Protective measures include partial weight bearing with crutches for 6 weeks and restricted knee motion for 12 weeks following surgery.

#### Control group (PRP)

The primary objective is to prevent excessive loading of the painful knee joint. Therefore, therapeutic exercises involving knee movements against maximal resistance during extension and flexion, as well as those incorporating rotational components or performed in high-stress positions, such as kneeling, are not recommended. Additionally, physiotherapy is not advised within the first 3 days following injection.

### Provisions for post-trial care {30}

Moreover, this treatment is not a ‘point of no return’ as it leaves open the options to perform all other treatments available.

### Outcomes {12}

#### Primary outcome

The primary outcome is the change in KOOS Pain subscale from baseline (day 0 or up to 1 month before) to 24-month follow-up visit and it will be assessed by the KOOS questionnaire.^26^ The KOOS questionnaire is a patient-reported outcome measure (PROM) questionnaire with five different subscales (pain, symptoms, activity of daily living, sports activities and quality of life (QoL).^26^ The KOOS Pain subscale demonstrates a high intraclass correlation coefficient (ICC) ranging from 0.80 to 0.97 in individuals with OA, indicating excellent reliability.^27^ The questionnaire is filled in online by a patient up to 2 weeks before the follow-up visit online. In exceptional cases, when the patient did not fill in the questionnaire before the visit online, this can be done in the clinic before the visit. The score is automatically calculated by the database according to the equation defined in the questionnaire.

#### Secondary outcomes

##### Questionnaires

The PROMs have been selected to address the core domains (pain, physical function patient global assessment and joint integrity) of patient-reported outcomes and based on their reliability and validity as well as their wide use in the clinic.^2628^,^32^

All questionnaires are completed online by the participants at baseline, after 6, 12 and 24 months.

- KOOS: in addition to the KOOS Pain subscale, we will evaluate the mean KOOS subscores at all timepoints (baseline, 6, 12 and 24 months). These data will give an indication of the recovery of the patient over time and potential differences between the two groups at defined time points.^26 27^
- Kujala Anterior Knee Pain Scale: this questionnaire is specifically designed and therefore, especially suited for patellofemoral pain. The results will allow a comparison between groups as well as verification of the results from the KOOS scores.^28^
- Western Ontario and McMaster Universities Osteoarthritis Index (WOMAC): the WOMAC was chosen as it is broadly used in the literature for OA and will allow comparison of results with external references.^27 29^
- European Quality of Life-5 Dimensions (EQ-5D-5L): this questionnaire is used to assess overall changes in patients’ QoL.^30 32^ The widely used questionnaire is especially important for health economic assessments and comparison with the literature data.
- Global Rating of Change: this widely used score assesses whether the patient condition has gotten worse, better or stayed the same and to quantify the magnitude of that change.
- Marx Activity Rating Scale: this activity-related PROM is widely used to assess the activity of a patient after treatments of the knee.^31^
- Visual analogue scale (VAS): the VAS is used to measure the pain of the patient.^33^

##### Non-responders

The number of non-responders was chosen as secondary outcome parameter because of the importance to assess how many patients will respond or not to the treatments. An improvement in KOOS Pain of 13 on a scale of 0–100 is considered a minimal clinically important difference; lower changes are typically associated with ‘non-responders’.^27^

##### Treatment failures

Treatment failure is defined as objective pathological clinical findings by the investigator directly correlated with subjective patients’ complaints resulting in a clinical deterioration of the subjective clinical outcome assessed by KOOS. Moreover, a patient is considered as treatment failure, when switching to an alternative surgical regenerative treatment or knee replacement. A clinical deterioration is defined as reduction in the KOOS score of >13 compared with baseline.

##### Radiological assessment

For the radiological evaluation of the OA knee joint, a comprehensive assessment of the whole joint with X-ray and MRI combining morphometric analysis, semiquantitative scoring and compositional measurements in several subregions of the knee joint is mandatory. MRI assessments were chosen as secondary outcome parameters as they allow monitoring of disease progress and assessment of maturation of the repair tissue. The latter is expected to provide an insight into the healing process and the possible regenerative nature of the treatment, to be correlated with mid-term and long-term clinical course of the disease. Moreover, MRI Osteoarthritis Knee Score (MOAKS) and MRI of Cartilage Repair Tissue (MOCART) scores provide an objective endpoint of high clinical relevance to assess cartilage regeneration, which is known not to be reached in the comparator group.^34^,^36^ Both MOCART and MOAKS scores have shown good inter-rater reliability and are widely used as standardised tools for assessing structural cartilage outcomes in clinical and research settings.^34 35^

The following parameters will be assessed

- MOAKS for radiological evaluation of OA at 6, 12 and 24 months.
- MOCART for radiological evaluation of repair tissue at 6, 12 and 24 months.
- OA grade according to Iwano and Kellgren/Lawrence classification at baseline, 6, 12 and 24 months.

##### Safety outcomes (biovigilance)

The study will evaluate the safety of the implantation of N-TEC by the number of serious adverse drug reactions or Suspected Unexpected Serious Adverse Reactions from baseline assessment up to 24-month follow-up assessment. Adverse events/adverse drug reaction will be graded according to severity, expectedness and relationship to trial treatment and reported according to the regulations.

### Other outcomes

#### Physiotherapeutic assessments

The two physiotherapy assessments Y-Balance Test and One Leg Hop Test are used as objective criteria for the function of the knee joint in terms of lower limb strength and dynamic balance before the intervention, after 6, 12 and 24 months.^37 38^ Both assessments are widely used in clinical settings and have demonstrated good test–retest reliability in healthy individuals, with ICC values ranging from 0.85 to 0.88 for the Y-Balance Test^39^ and from 0.94 to 0.96 for the One-Leg Hop Test.^40^

The assessments are carried out by the treating therapists, who have previously received online training and written instructions.

#### Activity tracker

During the study, Xiaomi Smart Band 8 activity trackers (Xiaomi, Haidian, Beijing, China) will be worn by patients for 1 week prior to each assessment time point (baseline, 6, 12 and 24 months) to determine their activity level and assess a potential correlation between physical activity and clinical outcomes (eg, pain levels in more vs less active patients). The activity trackers will record the total number of steps taken over each 1 week period as well as the average number of steps per day. This correlation may help in the interpretation of clinical outcomes.

#### Health economic outcomes

The condition-related costs over a 24-month period will be assessed from the perspective of a healthcare payer as well as from a societal perspective, including the costs associated with lost productivity. Quality-adjusted life years will be calculated over the 24-month period. The incremental cost-effectiveness at 24 months will be expressed as the cost per quality-adjusted life year gained, from both the healthcare payer and societal perspectives.

On a self-designed questionnaire, patients will report healthcare utilisation (medical resource use) and professional activity (productivity) at baseline (covering 6 months retrospectively) and at each follow-up time point (covering the time since the previous follow-up). Medical resource use and costs accrued at the treating medical centre will additionally be collected on the basis of administrative/financial data, from a subset of centres.

### Participant timeline {13}

Table 1 provides an overview of the participant timeline, from the first visit to the last follow-up measurement, including all assessments that are conducted.

**Table 1:** Participant timeline

Study periods	Screening	Treatment			Follow-up			
		N-TEC		Control (PRP)				
Visit	1	2a	2b	2	3	4	5	6
Time (hour, day, week, month)	≤ 6 m	Day −32	Day 0	Day 0, 7,14	6w	6m	12m	24m
Day	≤ 180	- 32	0	0, 7, 14	42	180	360	720
Acceptable time delay		0d	1d	2d	2w	4w	6w	8w
PIC	x							
Medical history	x							
Inclusion/exclusion criteria	x							
Laboratory tests (serology)	x	x						
Pregnancy test	x							
MRI*	x	(x)				x	x	x
X-ray*	x	(x)				x	x	x
Questionnaires†	x	(x)				x	x	x
EQ-5D-5L	x	(x)	x		x	x	x	x
Health economic data	x				x	x	x	x
Physiotherapy tests	x					x	x	x
Harvesting of nasal cartilage biopsy		x						
Surgery (implantation)			x					
PRP injection				x				
Clinical examination	x	x	x	x	x	x	x	x
Adverse events‡	x	x	x	x	x	x	x	x
Physiotherapy§			x	x	x	x		

* MRI and X-ray must be repeated if they were not performed during screening or were conducted more than 6 months before treatment (day 0).

† Questionnaires must be repeated if completed more than 1 month prior to treatment (day 0).

‡ Recording of the adverse events is a permanent process independent of specific visit.

§ Physiotherapy sessions will be performed continuously as prescribed by surgeon independent of specific visit. Physiotherapy is performed for 18 weeks (4.5 months) but can be prolonged if medically indicated.

EQ-5D-5L, European Quality of Life-5 Dimensions; N-TEC, nasal chondrocyte tissue-engineered cartilage; PIC, patient informed consent; PRP, platelet-rich plasma.

### Sample size {14}

The sample size was estimated assuming that the data will be analysed in a linear model with treatment as the variable of interest and KOOS at baseline as a covariate. It is assumed that patients will be randomised 1:1 (N-TEC:PRP). Based on published data, we assumed that the SD of the KOOS is 17.2 (pooled SD in both arms at baseline, number of patients: 183).^41^ Furthermore, we assumed that patients treated with PRP gain 10 points, whereas patients with N-TEC improve 25 KOOS pain units from baseline to 24 months. Recruiting a total of 75 patients allows to estimate a difference in the change in KOOS between the intervention arm and the control arm of 15 points with a power of over 90% and a two-sided alpha of 5%, assuming a dropout rate of 20% (15 patients).

### Recruitment {15}

Patients attending for normal consultation at the nine clinical centres will be screened for participation. If diagnosed with PFOA grade 1–3 according to Iwano classification patients will be informed of the trial and asked to participate. There is no advertisement for recruitment since no healthy volunteers are eligible for the trial. No compensation or payment will be given to the participants.

### Sequence generation {16a}

Patients will be randomly assigned either to injection of PRP or N-TEC implantation. The randomisation procedure will be implemented by the Clinical Data Centre of the Department of Clinical Research of the University Hospital Basel into the Clinical Data Management System (CDMS) REDCap^®^ per list. Randomisation will be stratified by study centre, with patients having a 50% probability to be randomised to the specific arm. In case the within centre imbalance between study arms will be equal to or larger than three, a ‘biased coin’ will be implemented such that patients will have an 80% probability to be allocated to the group that would minimise the imbalance between arms. This will reduce the imbalance within centre while ensuring allocation concealment to prevent selection bias in this open label study.

### Concealment mechanism {16b}

The assignment is not secret and is communicated to the person taking part, the doctor and the persons providing follow-up treatment.

### Implementation {16c}

Participants will be screened and enrolled by the respective principal investigator during their usual medical consultation in nine different centres (six in Switzerland, two in Germany, one in Croatia). Allocation to treatment groups is computerised.

## Assignment of interventions: blinding

### Who will be blinded {17a}

The study is not blinded as blinding of patients is not possible for ethical reasons (injection vs surgery). Also, assessors of MRI/X-ray cannot be blinded, as an experienced person will detect the implantation of the graft.

### Procedure for unblinding if needed {17b}

Not applicable, because the design is open label.

## Data collection and management

### Plans for assessment and collection of outcomes {18a}

Study data will be captured via the online CDMS REDCap^®^, based at the IT Department of the University Hospital Basel. The questionnaire as a measure for the patient- reported outcomes will be included in the electronic CRF to be filled in by the patient before the visit or directly at the site online or on paper.

All investigators directly involved in the patients’ information about the study, study intervention and follow-up will have to provide the certificate of Good Clinical Practice (GCP) training and/or follow additional national and local requirements on investigator qualification.

All outcomes are evaluated at baseline, and at 6-month, 12-month and 24-month follow-ups. Furthermore, the EQ-5D-5L and health economic data will be collected at the clinical visit scheduled for 6 weeks post-intervention.

### Plans to promote participant retention and complete follow-up {18b}

Data will be collected until the time of withdrawal using the questionnaires and MRIs at the time of follow-up. No data will be collected afterwards. A final medical visit is recommended for the safety of the patient after withdrawal (minimum 1 year after treatment).

### Data management {19}

Study data will be captured via the online CDMS REDCap^®^, based at the IT Department of the University Hospital Basel. The data collected is entered into the study eCRF. An audit trail will maintain a record of initial entries and any changes made; time and date of entry and username of person authorising entry or change. For each patient enrolled an eCRF must be completed. The principal investigator and coinvestigator at the study site will be responsible for assuring that the data entered into the eCRF are complete, accurate and that the entry and updates are performed in timely manner. If a patient withdraws from the study, the reason must be noted on a dropout form of the eCRF.

### Confidentiality {27}

Data will be collected only from patients who have given their written consent to our study. Their medical records will be handled confidentially. Only members of the study team, monitors and the respective authorities (competent authorities) will have access to the data. An enrolment log will be kept at each site as well as a code list to link the patient’s name with their patient ID. Documents will be stored according to the hospital regulations, but for a minimum of 30 years.

### Plans for collection, laboratory evaluation and storage of biological specimens for genetic or molecular analysis in this trial/future use {33}

Consent of the patient has to be obtained for the use of excessive cells for research purposes. Excessive cells not used for the generation of the implant will be stored in the research laboratories in Basel and Würzburg and used for research to deepen knowledge in the diseases. All biological materials given to the lab will be encoded. Only the principal investigators have access to the code. No connection is made between the biological material and patient data.

## Statistical methods

### Statistical methods for primary and secondary outcomes {20a}

The primary analysis will be based on the per-protocol estimand. Follow-up (change from baseline to 24 months) KOOS Pain values will be compared between the treatment arms using a linear regression model including baseline values as covariate (analysis of covariance (ANCOVA)) and centre as fixed effect. The model-based estimate of the treatment effect of tissue implantation as compared with plasma injection together with its 95% CI and p value will be presented. At the moment, the number of patients with a recurrent event is not assessable. Depending on this number and the distribution between the treatment arms, the per-protocol estimand may not be conservative. Therefore, the main estimand will be presented with the intention-to-treat estimand, and the number of intercurrent events will be listed by treatment arm. Possible bias due to intercurrent events will be discussed together with the results.

For demographic baseline variables, summary statistics will be presented. Continuous variables will be summarised by mean and SD in case of an assumed normal distribution or by median and IQR otherwise. Binary and categorical variables will be summarised by frequencies and percentages. KOOS Pain scores at 6 and 12 months as well as further KOOS subscales will be analysed in a similar fashion as the primary outcome namely based on ANCOVA, with the outcome as the dependent variable, the treatment arm as predictor and the baseline outcome value as covariate. The estimated, baseline-adjusted treatment effect of tissue implantation as compared with plasma injection will be reported together with its 95% CI. In case there are centres with fewer than five patients, these will be pooled with the next largest centre. Furthermore, summary statistics will be presented for the values at each assessed timepoint according to treatment arm, with outcomes summarised by mean and SD in case of an assumed normal distribution and by median and IQR otherwise. We will display the time course of the outcomes by appropriate graphics. Treatment failures will be summarised by frequencies and percentages. The proportion of patients with deterioration and non-response will be shown according to Iwano class.

### Interim analyses {21b}

No interim analysis is planned.

### Methods for additional analyses (eg, subgroup analyses) {20b}

No subgroup analysis is planned.

### Methods in analysis to handle protocol non-adherence and any statistical methods to handle missing data {20c}

Multiple efforts will be undertaken to retrieve missing data. No missing values are anticipated in the baseline characteristics. For the primary endpoint, the pattern of missing data will be systematically assessed. In the event of death, missing data will be assumed to be completely at random and will not be imputed. In cases of loss to follow-up, data will be assumed to be missing at random, and multiple imputations using chained equations will be considered.

### Plans to give access to the full protocol, participant level data and statistical code {31c}

Information on the study will be disseminated through websites of the hospitals or newsletters designed with the patient board of the study. The publication of the results is planned through submission of papers to peer-reviewed journals. In addition, the results will be communicated at national and international conferences and seminars as well as through presentations for lay persons. Study results will be published in the registered databases.

## Oversight and monitoring

### Composition of the coordinating centre and trial steering committee {5d}

It is a multicentre study with the University Hospital Basel as the leading centre. The following persons and committees are involved:

The sponsor-investigator also acts as the principal investigator and is responsible for coordinating the clinical trial across participating partners, ensuring harmonisation of procedures in addition to fulfilling all other sponsor and investigator duties. The Sponsor’s institution will also provide regulatory oversight and support for the trial submission and conduct. The principal investigators are responsible for the conduct of the clinical trial in accordance with GCP regulations and ICH-guideline E6 (R2). They are involved in the design and planning of the clinical trial and are responsible for all trial-related medical decisions at their site. Furthermore, they have the responsibility of training all further staff involved in the performance of the clinical trial at their site and reporting of all serious adverse events (SAEs) to the Sponsor-Investigator. The steering committee, consisting of the patient board and principal investigators, will convene one to two times per year, depending on the needs and phase of the study’s development. The patient advisory board consists of two patient representatives of patient organisations related to OA field and one patient previously treated with N-TEC.

The power analysis for the study has been conducted by the Department of Clinical Research at the University Hospital Basel. Serological tests will be carried out by the laboratories at the designated hospitals across the clinical sites. Monitoring will be performed by the Department of Clinical Research of the University Hospital Basel for all clinical sites, except Croatia, where monitoring will be carried out by Smart Medico d.o.o in coordination with the Department of Clinical Research at of the University Hospital Basel. The graft will be manufactured in GMP-compliant facilities in Germany at the University Hospital Würzburg and Switzerland at the University Hospital Basel, with a manufacturing authorisation from Government of Upper Franconia, Ansbach, Germany in consultation with the Paul-Ehrlich-Institute (NCA) or Swissmedic, Switzerland, depending on the manufacturing site.

### Composition of the data monitoring committee, its role and reporting structure {21a}

The monitoring will be performed by the Department of Clinical Research of the University Hospital Basel for all centres, except Croatia. A monitoring plan, describing in detail the documents and data to be monitored, has been established to guarantee a harmonised monitoring. An initiation visit is carried out before the start of the trial, interim visits and a close-out visit at the end of the trial are foreseen for each site. All source data and documents will be accessible to monitors and questions arising from monitoring will be answered by the participants of the audit from the site.

### Adverse event reporting and harms {22}

During the entire duration of the study (category C), all adverse events (AE) and all SAEs are collected, fully investigated and documented in source documents (patient’s records) and CRFs. Study duration encompasses the time from when the participant signs the informed consent until the last protocol-specific procedure has been completed and includes a safety follow-up period of up to 2 years.

### Frequency and plans for auditing trial conduct {23}

The responsible ethics committees, authorities and the sponsor have the right to and may inspect the study and/or manufacturing site at any time prior to, during or after the clinical conduct. There will be no audits in addition to the monitoring and inspections. Study documentation and the source data/documents are accessible to auditors/inspectors (also CEC and CA) and questions will be answered during inspections. All involved parties must keep the participant data strictly confidential.

### Plans for communicating important protocol amendments to relevant parties (eg, trial participants, ethical committees) {25}

In case of important protocol modifications (eg, changes to eligibility criteria, outcomes, analyses), relevant parties (eg, investigators, CEC, competent authorities, trial registries, regulators) will be notified within 5 days. Under emergency circumstances, deviations from the protocol to protect the rights, safety and well-being of human subjects may proceed without prior approval of the sponsor and the CEC/CA. Such deviations shall be documented and reported to the sponsor and the CEC/CA as soon as possible.

### Dissemination plans {31a}

The study was registered in an online trial registry clinicaltrials.gov. Information on the study will be disseminated through websites of the hospitals or newsletters designed with the patient board of the study. The study protocol will be published as an open access version. The publication of the results is planned through submission of papers to peer-reviewed journals. In addition, the results will be communicated at national and international conferences and seminars as well as through presentations for lay persons. Study results will be published in the registered databases, if applicable.

## Discussion

The aim of this study is to evaluate the use of N-TEC in comparison with PRP as a standard treatment option for patients with isolated PFOA. Safety, pain, function and structural outcomes of both treatments are compared. In addition, the study is complemented with a health-economic evaluation to establish the intervention’s value for money and impact on productivity in working age individuals.

Currently, no regenerative treatment options exist for PFOA. This study is the first RCT to evaluate the efficacy of N-TEC and the first to investigate a regenerative intervention in this condition. A previous study demonstrated promising results for N-TEC in the treatment of focal chondral lesions of the knee joint.^42^ Furthermore, its safety and feasibility were confirmed in case studies with OA^43^ as well as pilot patients treated under temporary authorisation in Switzerland suffering from PFOA (unpublished results).

A notable strength of this study is the combination of both subjective and objective outcome measures. In addition to PROMs, the study design includes objective criteria such as radiographic and MRI assessments, activity monitoring and standardised physiotherapeutic evaluations. Although postoperative physiotherapy is delivered at various locations, all participating physiotherapists receive individualised training in the standardised rehabilitation protocol. This ensures a uniform application of core treatment principles, while still allowing for patient-specific adaptations.

Nevertheless, the study has certain limitations. Due to the inclusion of a non-surgical comparator, blinding of participants is not possible, which may introduce bias. Also, a surgical approach may be seen by some patients as potentially having greater benefit, which may also introduce bias in the patient self-reported outcome.

### Trial status

Recruitment started on 6 June 2024. The planned duration of recruitment after the start is 2 years, and the last follow-up measurement takes place 2 years after the last intervention.

### Availability of data and materials {29}

The complete dataset is then exported and transferred to the study statistician as well as the principal investigator through a secure channel. A suitable subdataset is transferred to the study health economist, following the same principles as for the statistical analysis. The exported data will be archived for 10 years by the principal investigators in the respective centres.

### Ethics and dissemination

All centres involved in the implementation of the intervention have obtained approval from their respective competent ethics committees. This includes approval from the following ethics committees: Ethics Committees of North-Western and Central Switzerland (EKNZ): 2024–00075 (associated ethical committees: Cantonal Ethics Committee Bern, Cantonal Research Ethics Commission Geneva (CCER), Cantonal Ethics Committee Ticino, Cantonal Ethics Committee Zurich). The EKNZ covers several cantons in Switzerland, including Basel. The site in Lugano falls under the Cantonal Ethics Committee, Ticino. Ethics Germany, according to CTIS: 2023-508640-21-00 (Medicinal Ethical Commission of the Julius-Maximilians-University Wuerzburg, Ethical Commission of the Albert-Ludwigs-University Freiburg) and Central Ethical Committee Croatia, Republic of Croatia Ministry of Health: 2023-508640-21-00. The Swissmedic reference number is 701788.

Participation in the study is voluntary, and all participants are required to provide written informed consent. Participants may withdraw their consent at any time without providing a reason and without any negative consequences. Data will be collected only from patients who have given their written consent to our study. Their medical records will be handled confidentially. Only members of the study team, monitors and the respective authorities (ethical committees, competent authorities) will have access to the data.

The study was registered in an online trial registry clinicaltrials.gov. Information on the study will be disseminated through websites of the hospitals or newsletters designed with the patient board of the study. The study protocol will be published as an open access version. The publication of the results is planned through submission of papers to peer-reviewed journals. In addition, the results will be communicated at national and international conferences and seminars as well as through presentations for lay persons. Study results will be published in the registered databases, if applicable.

## Supplementary material

10.1136/bmjopen-2025-106140 online supplemental material 1
