# Supplementary material for: Treatment of patellofemoral osteoarthritis with nasal chondrocyte-based engineered cartilage implantation in a randomised, controlled, multicentre phase II clinical trial: protocol for a randomised controlled trial
Source: BMJ Open. 2025 Aug 21;15(8):e106140. doi: 10.1136/bmjopen-2025-106140 (PMC12374661; doi:10.1136/bmjopen-2025-106140)
Supplement: online supplemental material 1 [file bmjopen-15-8-s001.pdf]

Request to participate in medical research

Study title: Treatment of patellofemoral osteoarthritis with nasal chondrocyte-based engineered cartilage implantation in a randomized, controlled, multicenter phase II clinical trial

Layman understandable title: Treatment of osteoarthritis behind the kneecap with cultivated nasal cartilage cells in a controlled, multicenter clinical trial

Dear Madam/Sir

Thank You for your interest in taking part in this clinical study about patellofemoral osteoarthritis treatment. We would like to inform you here about the details of the study and ask you whether you would like to participate. Indeed, before a new drug or a new treatment method can be used by doctors, research must be carried out to find out how this drug or treatment method works. We call such research projects **clinical studies**.

In this study, we want to find out what effect the test substance N-TEC (a cartilage-like tissue cultivated in laboratory from the body's own nasal cartilage cells), which is implanted into the knee joint defect, has on the healing of patellofemoral osteoarthritis or whether it can prevent the progression of the disease. You suffer from patellofemoral osteoarthritis and you are supposedly already undergoing treatment for it. Therefore, we are asking you if you would like to participate in this study.

Your participation is on a voluntary base. The following **patient information and informed consent form** will help you to decide whether you wish to participate or not. You can ask any questions you have about participating in the study by **speaking with one of the investigators**. That is the name given to the clinicians who are responsible for the study and who will look after you as part of this study. If you want to participate, please **sign the informed consent form** at the end. Your signature confirms that you have read and understood the patient information. If there is anything you do not understand, please ask the investigators.

The patient information and informed consent consists of 4 parts:

Part 1 The most important in a nutshell
Part 2 Detailed information about the study
Part 3 Data protection and insurance coverage
Part 4 Informed consent

When reading **part 1**, you will get an overview of the study. In **part 2** we explain the whole process and background of the study in details. **Part 3** contains information on data handling and insurance protection. With your signature at the end of the document, **part 4**, you confirm that you have understood everything and agree to participate.
This study is organized by the University Hospital Basel. This institution is called the "sponsor". The sponsor is responsible for the study, manages and finances it.

Within the framework of this study, the investigators responsible for you are:

Name PD Dr. Marcus Mumme
Address Universitätsspital Basel
Orthopädie und Traumatologie
Spitalstrasse 21, 4031 Basel, Schweiz
Telephone +41 61 556 5885
+41 61 265 25 25 (24h Erreichbarkeit)
e-mail: Marcus.Mumme@usb.ch

Name Dr. Györzö Lehoczky
Address Universitätskinderspital beider Basel
Orthopädie
Spitalstrasse 3, 4031 Basel, Schweiz
Telephone +41 61 704 2615
+41 61 704 12 12 (24h Erreichbarkeit)
e-mail: gyoezoe.lehoczky@unibas.ch

Part 1:
The most important in a nutshell

1. Why are we conducting this study?

In the case of osteoarthritis of the patella, standard therapies include physiotherapy, injections of hyaluronic acid or platelet-rich plasma from the patient's own blood, or artificial joint replacement with a (partial) knee prosthesis. These are intended to provide relief of pain and improved knee function.

In this study, we are investigating how the test substance N-TEC works in the treatment of knee osteoarthritis and whether it is effective and well tolerated. In **chapter 4** you will learn more about the scientific background of the study.

2. What do you have to do, if you participate?

Participation in this study will last 2 years for you, including the follow-up period. We will invite you for four study visits. Clinical follow-up appointments are also part of your general treatment and will also take place independently of your study participation. In addition, radiological controls with X-ray and MRI will be performed after 6 months, 1 and 2 years, these are only part of the study. A clinical control appointment lasts about 15-20 minutes, and if X-ray and MRI examinations are performed, you must allow 30-60 minutes of additional time. The number of appointments is indicated in the **chart in Chapter 5**.

If you decide to participate, you will be randomly assigned to one of two groups. You will belong to either the experimental group or the control group. In the experimental group, you will be treated with the test substance N-TEC. This involves taking a small piece of cartilage (biopsy of 7x7mm size) from the nasal septum 4 weeks prior to implantation and implanting the cultured cartilage tissue into the knee (open knee operation). In the control group, you will receive platelet-rich plasma (PRP) from your own blood. For this, blood will be taken from you, prepared and injected back into the joint with a syringe (injection).

In **Chapter 5**, you will learn more about the process and procedure of the study.

3. What is the benefit and the risk associated with participation?

Benefit

If you participate in this cartilage regeneration treatment, you may benefit from the following:

In the most favorable case, N-TEC heals in the cartilage defect in the knee joint and supports the formation of articular cartilage comparable to healthy cartilage. This would be expected to lead to an improvement in pain and functionality of the knee joint. However, it is not known if an improvement in pain, knee joint mobility and functionality can actually be achieved or if healing or progression of osteoarthritis can be prevented. The study is randomized, meaning the patient is randomly assigned either to the study group or to the control group. The split ratio is 50:50, meaning that half of the patients receive the investigational product, and the other half the treatment with platelet-rich plasma (which is the control treatment).

Your participation contributes to current medical research and can therefore help new therapeutic approaches to be implemented in practice so that future patients can also benefit.

Risk

The test susbtance N-TEC is not yet approved in Switzerland.

You may experience side effects when you are treated with the study drug N-TEC. We may not yet know all the risks and side effects of the test substance. So far, the following risks and side effects are known:

- Dislodgement of the implant with possible need for follow-up surgery.
- Pain and swelling associated with surgery
- Infection of the puncture, biopsy site or of the surgical wound

See **Chapter 6** for more information on risks and burdens.

Part 2:
Detailed information about the study

4. The scientific background of the study

4.1 Background: Why do we conduct this study?

Patellofemoral osteoarthritis is a common condition in younger and older people. When you have patellofemoral osteoarthritis, you typically suffer from pain and limited function of the knee joint. Patellofemoral osteoarthritis is usually treated with physical therapy and pain medications or injections of hyaluronic acid or platelet-rich plasma from the patient's own blood, or in advanced stages, artificial joint replacement with a (partial) knee prosthesis.

There is already research in humans for the treatment of patellofemoral osteoarthritis. Previous human studies have shown that treatment with hyaluronic acid or platelet-rich plasma can achieve some success in reducing symptoms. In studies completed to date for isolated cartilage defects involving more than 65 patients, the N-TEC test substance has shown that significant improvement in pain and symptoms can be achieved and the cartilage surface in the joint can be rebuilt. However, only a few patients suffering from osteoarthritis have been treated with N-TEC.

In this study, we are therefore investigating whether the test substance N-TEC is effective and well tolerated in knee osteoarthritis. The test substance N-TEC contains autologous nasal cartilage cells and the cartilage matrix produced by these cells as the active ingredient. The test substance N-TEC has not yet been approved in Switzerland. Only when the efficacy of the test substance N-TEC will be scientifically investigated and proven it can be approved in Switzerland and used against patellofemoral osteoarthritis.
We are also investigating whether the implantation of the test substance can prevent the progression of osteoarthritis or even achieve regeneration.

4.2 Design of the study: What do we do?

In our study, participants are randomly assigned to one of the groups. This is important to obtain reliable results of the study. This is called randomization. Each group receives a different treatment. In our study, there are two groups:

- **Group 1** (experimental group) gets the test substance implanted into the joint defect (open knee surgery).
- **Group 2** (control group) receives the standard treatment with platelet-rich plasma (PRP) from the patient’s own blood (in an injection form).

Half of the patients are assigned to group 1 and the other half to group 2, so there is a 50% chance that you will receive the investigational product and a 50% chance that you will receive the control, standard treatment. Randomization allows us to assess more objectively how well the treatment really works and whether it is safe.

4.3 Regulations on scientific research with human subjects

We conduct this study in accordance with the laws in Switzerland (Human Research Act, data protection laws). We also comply with all internationally recognized guidelines. The responsible ethics committee and Swissmedic have reviewed and approved the study.

Our study is an international study. This means that there are 7 participating clinics in Switzerland, 2 clinics in Germany and 1 clinic in Croatia.

A description of this study can also be found on the website of the Federal Office of Public Health at www.kofam.ch under the BASEC registration number 2024-00075.

5. Course of the study

5.1 What do you have to do, if you participate in the study?

Participation in the study is voluntary and lasts 2 years. You must adhere to the schedule (→ Chapter 5.2) and also to any instructions given by your investigator.

You must inform your investigator

- if your health condition changes, e.g. if you get worse or if you have new symptoms; this also applies if you drop out of the study early (→ chapters 5.3 and 5.4);
- if you receive concurrent treatment and therapy from another doctor and take other medications. Please list all medications, including those you purchased yourself for which you do not need a prescription, or herbal teas, herbal remedies, and alternative medicine medications (homeopathy, spagyric, etc.).

You must also observe the following:

- You must effectively prevent the occurrence of pregnancy during participation (→ chapter 5.5).

5.2 What happens at the appointments?

For the intervention group, a 7x7mm piece of cartilage is removed from the nasal septum 4 weeks before implantation, to produce the test substance ("N-TEC"). The harvesting is performed under local anesthesia. The piece of cartilage is sent to a special laboratory in Switzerland (Basel) or Germany (Würzburg) for further processing. First, the cartilage cells are extracted from the tissue and multiplied. After two weeks, sufficient cells are available, which are then applied to a collagen membrane. Within the next two weeks, the cartilage cells form cartilage-like tissue on the membrane, which is then implanted into the knee joint in an operation.

For the control group, patients will come directly for injection of platelet-rich plasma (PRP), 3 times in a row with weekly distances. This occurs with first retrieval of 15ml own blood, preparation of the PRP concentrate and injection in the knee within 30 minutes from withdrawal.

During the course of your participation, you will come to us four times for a study visit after the treatment (cartilage harvesting and implantation or 3 x injection of platelet-rich plasma). The clinical control appointments are also part of your general treatment and also take place independently of your study participation. In addition, radiological controls with X-ray and MRI will be performed at 6 months, 1 and 2 years, these are part of the study only. A clinical control appointment takes about 15-20 minutes, if X-ray and MRI examinations are performed, you will need to allow 30-60 minutes of additional time. The sequence of appointments is shown in the figure below.

At all appointments, we do the following:

- We answer your questions.
- We ask you questions about your health status.

- We perform a clinical examination.
- We ask about any side effects or other medical conditions

At individual appointments we also perform:

- X-ray examinations, i.e. we use X-rays to image the bones of the knee in order to examine the size of the joint space.
- Magnetic Resonance Imaging (MRI), which means that we image the cartilage parts of the knee to examine the repair of the defects and the healing of the cartilage piece into the underlying bone.
- Questionnaires answered by the patient to study the patient's subjective impression of the effectiveness as well as data for cost effectiveness for the treatment.

Through these tests we can see how well the test substance works and whether it is safe.

The schedule on this page shows all appointments. The **general examinations** are marked with **a gray check mark** (✓). The additional examinations are marked with a **plus sign** (+). Only these examinations therefore represent an additional time expense for you.

Study course: General and additional examinations

**Study visit / appointment**	**1**	**2**	**3**	**4**
**Time point**	**6 weeks**	**6 months**	**12 months**	**24 months**
**Duration (hours)**	**0.25-0.5**	**0.5-1**	**0.5-1**	**0.5-1**
Clinical examination	✓	✓	✓	✓
Questionnaires		+	+	+
x-ray		✓	+	+
MRI		+	+	+
Adverse Events	+	+	+	+

We arrange the appointments together with you. You will receive an exact overview of the appointments. Appointments cannot simply be postponed. We ask you to inform us quickly if you nevertheless have to postpone an appointment for important reasons.

5.3 When does your participation in the study end?

For you, participation lasts 2 years and ends after the fourth appointment. You can also terminate your participation earlier at any time (→ chapter 5.4). You do not have to explain why you no longer wish to participate. If you wish to end your participation earlier, please talk to your investigator.

Even if you terminate your participation early, we will continue to treat and care for you medically as well as possible according to current standards (→ chapter 5.4 for alternative treatment options). In this case, we will perform a final examination for your safety.

If you stop the study earlier, we ask you to continue to inform your investigator if your health status changes, e.g. if you get worse or if you have new complaints. If your participation ends early, we will still evaluate the data and samples collected up to that point (e.g., MRI or X-ray images and questionnaires) for the study.

We may also need to ask you to end the study early. This is the case, for example, if there are medical reasons why continuing the treatment would endanger your health. Also, if you withdraw consent or do not take the necessary measures as described in the patient information and consent form, or refuse the visits that are necessary to assess the safety and effectiveness of the treatment. Furthermore, we must exclude you if the test substance is contaminated during manufacture or if manufacture fails.

5.4 What happens, if you do not want to participate?

Even if you do not participate in this study, we will treat and care for you medically in the best possible way according to current standards. If you do not wish to participate in the study, your investigator will advise you in discussion about alternative treatment options.

5.5 Pregnancy

The test substance N-TEC is not yet approved. It may be dangerous and harmful to an unborn child. Therefore, women in this study must not get children while participating. You will discuss your questions with your investigator.

For women, who could become pregnant

You must not become pregnant during your participation in the study. You must inform your partner(s) that you are participating in this study. Before starting the study, you will perform a pregnancy test. No further such tests will be carried out during the study. If you are breastfeeding, you may not participate.

You must use a double contraceptive method while participating in the study:

1. a preparation that suppresses ovulation either as a tablet ("pill" / "mini-pill"), injection, rod under the skin, patch or vaginal ring in combination with a condom or
2. condom in combination with copper or hormonal intra uterine device (IUD).

You do not have to use these contraceptive methods after the end of the study. If you still become pregnant during the study, you must tell your investigator immediately. He will then talk to you and your partner about how to proceed.

6. Risk, burdens and side effects

6.1 What risks and burdens can occur?

There are risks and burdens in participating in this study, as with any medical treatment. Some risks we already know, others are still unknown. This uncertainty is not unusual in the study environment. You will find a list of the most common and most serious risks in **Chapter 6.2.** Many side effects are medically treatable. We will inform you of any new findings on risks and side effects during the study.
With a new test substance, it is possible that there are risks that we do not yet know.
The test substance N-TEC has already been used in more than 65 patients.
Implantation may result in the standard risks associated with surgery. These are explained in the hospital's standard explanation for surgery.
In addition, there are risks associated with the medical exams we do in this study. You might already be familiar with some of the examinations. You will find a list of these risks of the examinations in **Chapter 6.3**.

6.2 The most common and serious risks posed by the investigational drug

You can find information about the most common and serious side effects that we already know about.

We use the following descriptions for this:

Very often	We find the side effect in more than 10 people out of 100 (more than 10%).
often	We find the side effect in 1 to 10 people out of 100 (1%-10%).
Occasionally	We find the side effect in 1 to 10 people out of 1,000 (0.1%-1%).
rare	We find the side effect in 1 to 10 people out of 10‘000 (0.01%-0.1%).
Very rare	We find the side effect in less than 1 person out of 10‘000 (below0.01%).

Very common side effects are:
- Swelling or bruising in the knee (expected temporarily after implantation).
- Knee joint pain (expected temporarily after implantation)
- Bone marrow edema

Common side effects include:
- Joint effusion
- Joint swelling

Occasional but dangerous side effects are:
- none known

unknown frequency:

- Complete detachment of the investigational product
- Infection at the puncture site, harvesting site or surgical wound

6.3 Risks and burdens due to the examinations of the study

We do several medical examinations for this study (→ chapter 5.2). These examinations are proven procedures. Nevertheless, they can have risks and burdens, that is, they can be unpleasant or have undesirable side effects. In this study, there are the following risks and burdens:

- Blood collection: bruising, bleeding, or swelling at the injection site may occur. Rarely, infection may occur at the puncture site.
- Magnetic resonance tomography: Rarely, dizziness or claustrophobia may occur.
- X-ray: There is very little exposure to radiation.

7. Funding and compensation

This study is initiated by the sponsor University Hospital Basel and paid for by a research grant project from the Swiss National Science Foundation. This study is fully funded by this project. The test substance N-TEC is provided free of charge by the sponsor.

The participating researchers have no financial benefit in the conduct of this study.

If you participate in this study, you will not receive any money or other compensation.

There is no additional cost to you or to your health insurance by participating in this study.

The results of this study may help to sell a drug later. You will not be involved in this if you take part in this study.

8. Results of the study

There are results that affect you. These results will be communicated to you by your investigator. There are also incidental findings. Incidental findings are "accompanying results" that are not intended. These can be, for example, results of MRI or X-ray examinations and results of blood tests. We will inform you if these incidental findings are relevant to your health.

For example, we will inform you if we happen to find a disease that you do not yet know about and that we can treat. If you do not want to be informed, please discuss this with your investigator.

There are also the overall results of the study that come from the data from all participants. These include, for example, that we know more about effectiveness of implanting a tissue-engineered cartilage to treat patellar osteoarthritis (→ Chapter 4.1). These results do not directly affect you or your health. However, your investigator will be happy to give you a summary of the overall results of the study at the end of the study if you wish.

Part 3:
Data protection and insurance

9. Protection of data and samples

We protect your data (e.g. information such as blood pressure and pulse from your medical history) and your samples (e.g. your blood samples). There are strict legal regulations in Switzerland for the protection of data and samples.

9.1 Encryption of data and samples

Each study generates data from the examinations (e.g. blood values, MRI and X-ray images, as well as information about your health and from questionnaires). These data are documented. This is usually done electronically in large tables, the so-called "data collection sheets". All data is documented in encrypted form. "Encrypted" means that personal information is kept separate from the examination results. For this purpose, there is a list that identifies each person with a unique code. For example, your name, date of birth or place of residence do not appear directly in the data collection booklet. This list remains at the hospital for a period of 30 years. No one else gets this list.

At the end of the study, your data will be completely anonymized, at the earliest at the end of the legally prescribed retention period. This means that it will no longer be possible to identify you without disproportionate effort. Various measures are used for anonymization, including the destruction of the code and the list.

If we pass on data - to the sponsor or to specialists who carry out further examinations - then the data is always encrypted and your personal data is protected. This also applies if the data is passed on abroad. In this study, tissue samples from the nasal septum may be sent to the University Hospital of Würzburg in Germany to grow the cartilage implant. Only the absolutely necessary data are passed on and these are encrypted. The data required for production are stored for 30 years.

All samples (e.g. blood and tissue samples) are also always encrypted in this way. Your personal data is therefore protected when we send samples to be examined in the laboratory. Even in the laboratory, the data and samples always remain encrypted.

9.2 Safe handling of data and samples during the study

The sponsor, University Hospital Basel, is responsible for the safe handling of your data and samples from this study. He is responsible for ensuring that the applicable laws, e.g. data protection laws, are complied with. This also applies if (encrypted) data or samples are sent for investigations to countries where data protection laws are less favorable. This is how the sponsor of this study protects your data:

In this study, your data are collected and transmitted electronically. The data is stored on a server in Switzerland. Nevertheless, there is always a certain residual risk that strangers access your personal data (e.g. risk of "hacking").

It is often important that your family doctor shares data of your medical history with the investigator. This also applies to other physicians who treat you. By giving your consent at the end of the document, you allow this.

9.3 Safe handling of data and samples after the study

The sponsor remains responsible for the safe handling of your data and samples after the end of the study. The law requires that all study documents, e.g. the data collection forms, are kept for at least 20 years.

After a study is completed, the results are usually published in scientific journals. For this purpose, the results are reviewed by other experts. Your encrypted data must be forwarded to these experts. However, the data may not be used for new research purposes. Your separate consent would be required for this (see 9.4).

9.4 Further use of your data and samples in other, future studies

Your data and samples from this study - including genetic data - are very important for future research. Data and samples that have not already been completely consumed for this study may be reused for other studies. We need your separate consent to reuse your genetic data and samples. This is optional. Please read the additional consent form at the end of this document carefully and sign the consent if you would like your data and samples to support further research in the future. Even if you do not consent, you can still participate in the study.

9.5 Inspection rights during inspections

The conduct of this study may be subject to review. The review is carried out by authorities such as the responsible ethics committee or the regulatory authority Swissmedic or also by foreign regulatory authorities. The sponsor must also make such checks to ensure the quality of this study and the results.

For this purpose, a small number of specially trained persons are given access to your personal data and medical history. The data are therefore not encrypted for this review. The persons who see your unencrypted data are bound to secrecy.

10. Insurance

You are insured if you suffer damage as a result of the study - i.e. from the test substance. The procedure is regulated by law. For this purpose, the sponsor has taken out an insurance policy with HDI Global SE, Hardstrasse 201, 8005 Zürich. If you think that you have suffered damage as a result of the study, please contact your investigator or the insurance company directly.

In the case of damage caused by an approved drug used according to medical standards, e.g. platelet-rich plasma, or which would have occurred even if a usual therapy had been used, the same liability regulations apply as for treatment outside of a study. In such a case, the liability insurance of the hospital will cover the costs / compensation.

Part 4:
Informed consent

This consent consists of two independent consent forms:

- Informed consent for participation in this study PFOA II.
- Informed consent form for further use of data and samples from this study in encrypted form.

Please read this form carefully. Please ask us if there is anything you do not understand or if there is anything else you would like to know. Your written consent is required for participation.

Informed consent for participation in this study PFOA II

**BASEC-Number**	2024-00075
**Title of the study**	Treatment of patellofemoral osteoarthritis with nasal chondrocyte-based engineered cartilage implantation in a randomized, controlled, multicenter phase II clinical trial
**Layman understandable title**	Treatment of osteoarthritis behind the kneecap with cultivated nasal cartilage cells in a controlled, multicenter clinical trial
**Responsible Institution**<br>(Sponsor with Address)	University Hospital Basel<br>Spitalstrasse 21<br>4031 Basel<br>Switzerland
**Place of implementation**	University Hospital Basel
**Investigators at the study site**	PD Dr. Marcus Mumme
**Participant:**<br>Last name and first name in block letters:<br>Date of birth:	

- I have received oral and written information about the study from the investigator signing below.
- The investigator has explained to me the purpose, procedures, and risks of the study.
- I am voluntarily participating in the study.
- The investigator has explained to me what possible standard treatments are available outside of the study.
- I have had enough time to make this decision. I will keep the written information and receive a copy of my written informed consent.
- I can stop participating at any time. I do not have to explain why. Even if I stop participating, I will continue to receive my medical treatment. The data and samples collected up to that point will remain stored and will be analyzed as part of the study.
- If it is better for my health, the investigator can exclude me from the study at any time.
- I understand that my data and samples will only be passed on in encrypted form and sent abroad (Germany). The sponsor will ensure that data protection is maintained according to Swiss standards.
- In case of results and/or incidental findings that directly affect my health, I will be informed. If I do not wish to be informed, I will discuss this with my investigator.
- My family doctor may share data of my medical history that are important for the study with the investigator. This also applies to other doctors who are treating me.
- The responsible experts of the sponsor, the ethics committee and/or the medicinal products authority Swissmedic may view my unencrypted data for control purposes. All these persons are subject to the duty of confidentiality.
- I know that the institution University Hospital Basel has taken out an insurance policy. This insurance pays if I suffer damage - but only if the damage is directly related to the study.
- The residual tissue of the harvested cartilage piece from my nasal septum, the isolated cells and the remnants of the cultured cartilage may be further used for research purposes.

Place, Date	Last name and first name participant in block letters<br><br><br>Signature participant

Confirmation of the investigator: I hereby confirm that I have explained the nature, significance and scope of the study to this participant. I assure that I will fulfill all obligations related to this study according to Swiss law. If, during the course of the study, I learn of any aspects that could influence the participant's willingness to participate in the study, I will inform him/her immediately.

Place, Date	Last name and first name investigator in block letters<br><br><br>Signature Investigator

Declaration of consent for further use of data and samples in encrypted form

This consent does not concern you in the sense of personal participation in a study. "Further use" means that data and samples may be stored beyond the time of your participation in the study and used in encrypted form for further research. This can mean, for example, that a blood sample and corresponding laboratory values from you are statistically evaluated together with a large number of other values or that new investigations are carried out on them.

**BASEC-Number:**	2024-00075
**Title of the Study**	Treatment of patellofemoral osteoarthritis with nasal chondrocyte-based engineered cartilage implantation in a randomized, controlled, multicenter phase II clinical trial
**Layman understandable title**	Treatment of osteoarthritis behind the kneecap with cultivated nasal cartilage cells in a controlled, multicenter clinical trial
**Participant:**<br>Last name and first name in block letters:<br>Date of birth:	

- I allow my encrypted (genetic) data and samples from this study to be used for medical research. They will then be available for future research projects indefinitely.
- I understand that the samples are encrypted and the key is stored securely.
- The data can be evaluated within Switzerland and abroad and stored in a database here or abroad. The samples can be examined here or abroad and stored in a biobank. Research institutions abroad must adhere to the same data protection standards as those in Switzerland.
- I make a decision voluntarily and can revoke this decision at any time. If I withdraw, all of my data will be anonymized and my samples and (genetic) data will be destroyed. I simply inform my investigator and do not have to justify this decision.
- Normally all data and samples are evaluated together. If by chance a result is shown that is very important for my health, I will be contacted. If I do not want this, I will inform my investigator.
- I allow my data and samples to be anonymized and understand that in this case I **cannot** be informed about my personal results and cannot withdraw from the research project.

Place, Date	Last name and first name participant in block letters<br><br><br>Signature participant

Confirmation from the investigator: I confirm that I have explained to the participant the nature, meaning and scope of the further use of samples and/or genetic data.

Ort, Datum	Last name and first name investigator in block letters<br><br><br>Signature Investigator
